# A Little Experience Goes a Long Way: Chlormethine/Mechlorethamine Treatment Duration as a Function of Clinician-Level Patient Volume for Mycosis Fungoides Cutaneous T-Cell Lymphoma (MF-CTCL)—A Retrospective Cohort Study

**DOI:** 10.3389/fmed.2021.679294

**Published:** 2021-07-02

**Authors:** Christiane Querfeld, Theresa Pacheco, Bradley Haverkos, Gary Binder, James Angello, Brian Poligone

**Affiliations:** ^1^City of Hope Comprehensive Cancer Center, Duarte, CA, United States; ^2^Division of Dermatology, University of Colorado, Denver, CO, United States; ^3^Division of Hematology, University of Colorado, Denver, CO, United States; ^4^Helsinn Therapeutics US Inc., Iselin, NJ, United States; ^5^Rochester Skin Lymphoma Medical Group, Fairport, NY, United States

**Keywords:** cutaneous T-cell lymphoma, chlormethine gel, mechlorethamine, dermatitis, patient volume, treatment outcomes, mycosis fungoides

## Abstract

Topical chlormethine yields high response rates in mycosis fungoides cutaneous T-cell lymphoma with early discontinuation often attributed to skin reactions. We evaluated over 4,000 patients and found an association of clinician case volume with treatment duration and early discontinuation of chlormethine gel. The minority of clinicians with high patient volume markedly outperformed clinicians with only few patients on both outcome parameters, yet case volume as low as five patients seemed to mark a threshold for avoiding early discontinuation of treatment regimen.

## Introduction

Guidelines from the National Comprehensive Cancer Network (NCCN) recommend skin-directed therapies including topical chlormethine (mechlorethamine) as first-line treatment for early-stage mycosis fungoides cutaneous T-cell lymphoma (MF-CTCL) with localized or widespread skin disease. Chlormethine gel yields high response rates, particularly with treatment >3 months (1, 2). Early discontinuation is often attributed to skin reactions (1, 3). Treatment discontinuation as a result of non-compliance has previously been observed with ointment-based topical treatments such as chlormethine ointment or certain corticosteroids, mainly due to greasiness, but this appears to be less common with chlormethine gel (1, 2, 4, 5). We evaluated overall patterns of treatment and the association of clinician patient volume with early discontinuation and overall treatment duration for United States clinicians prescribing standardized chlormethine 0.016% gel formulation.

## Methods

### Ethics

Ethical review and approval was not required for this study in accordance with the local legislation and institutional requirements. Written informed consent for participation was not required for this study in accordance with the national legislation and the institutional requirements.

### Study Design

A retrospective cohort assessment was performed based on chlormethine gel dispensing records from the main specialty pharmacy that dispenses approximately 90% of chlormethine gel (>99% 1-month supply), representing most of the United States' utilization from October 2013 to April 2019. We excluded patients initiating treatment ≤100 days of data cutoff. Overall patient monthly discontinuation patterns were assessed, with early discontinuation defined as <3 months of treatment. When ≥4 prescribers treated an individual patient, patients of those prescribers were assigned to the treating institution or the most common prescribing clinician. This was rare, and usually involved residents under the supervision of faculty prescribers at teaching institutions. Other patients of those prescribers were similarly assigned to the treating institution or the most common prescriber. We calculated the average quantity of dispenses per patient as a surrogate for months of treatment duration. Prescribers were then grouped by number of patients treated with chlormethine gel in this period, and median treatment duration was calculated for each group. Kruskal–Wallis testing was performed on groups and logistic regression on early discontinuation. Variability was assessed by interquartile range (IQR).

## Results

We assigned 4,922 patients (55% male, median age 63 years among the 35.7% with known age) to 2,004 clinicians (Table 1). Across the full study population, the discontinuation rate was 33% for treatments 1–3; thereafter, rates averaged 16%. Patients receiving >1 dispense had 5 months median treatment duration (range 2–65).

**Table 1:** Patient demographics.

**Patient characteristic**	***N*/Value**	**% Total**
Female	2,210	44.9%
Coverage		
Medicare	1,172	23.8%
Commercial	2,751	55.9%
Other	248	
28- to 31-day supply	4,870	98.9%
Birth year known	1,758	35.7%
Median age	63	

**Figure 1:**
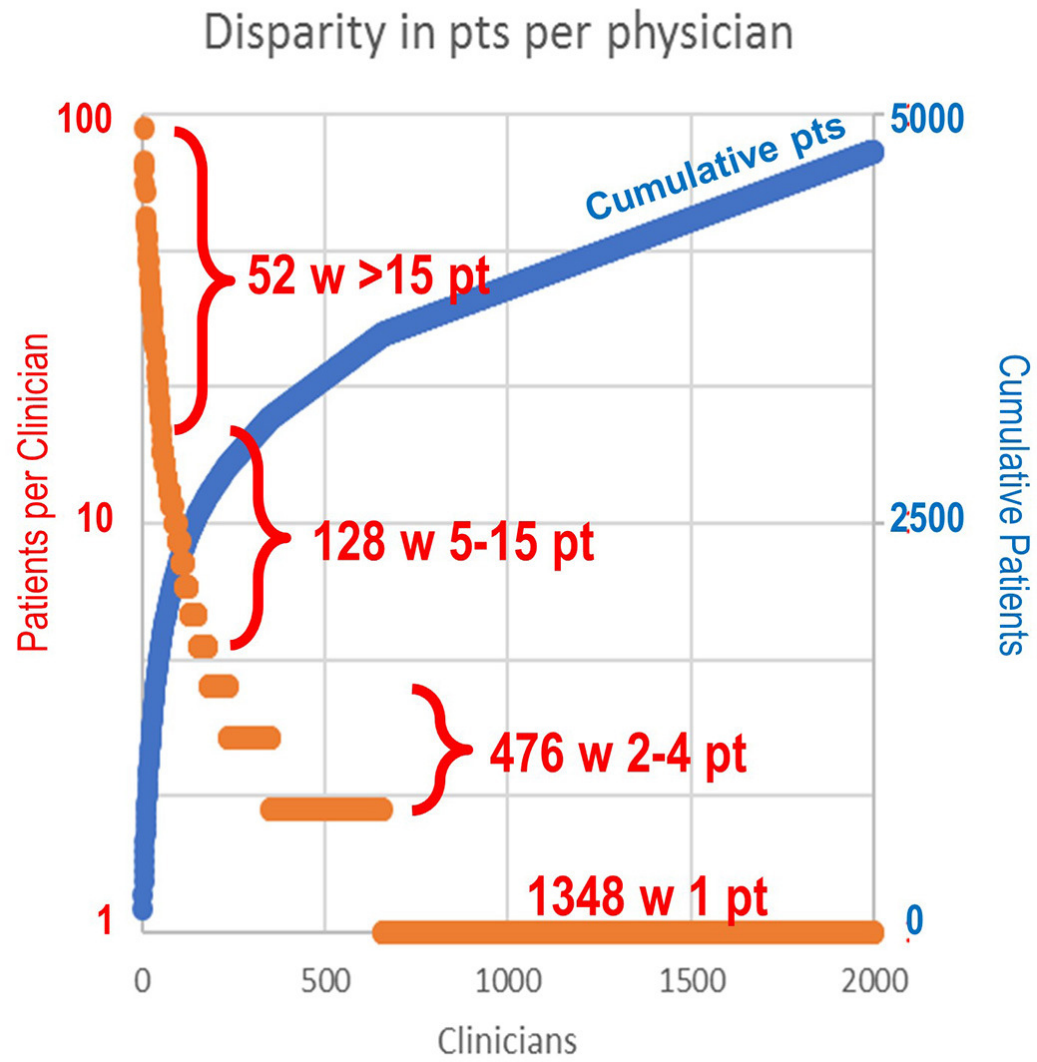
Range of patient volume per clinician. The 52 clinicians (2.6%) with >15 patients each (mean 41.3) treated 44% of total patients. The next 128 clinicians each treated 5–15 patients. A total of 476 clinicians treated two to four patients each. A total of 1,348 clinicians had only a single patient.

**Figure 2:**
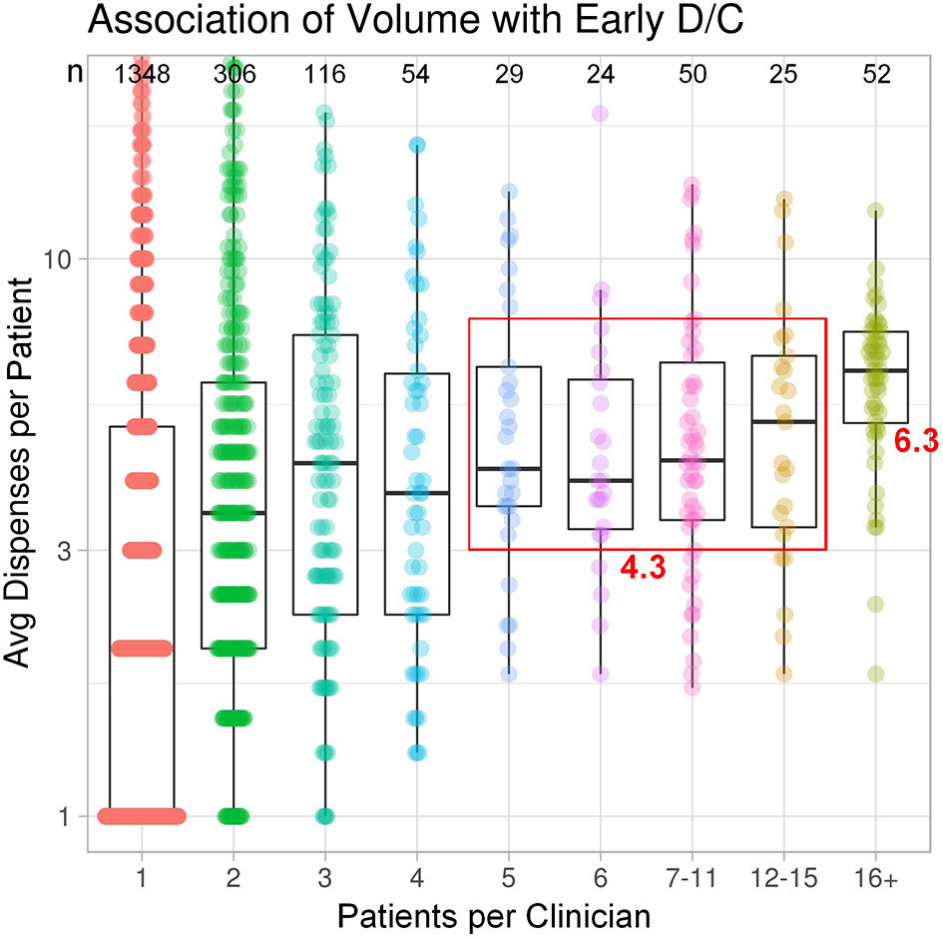
Treatment variability and early discontinuation increased with decreased patient volume. Improved adherence to treatment was noted for clinicians with higher patient volume.

In terms of patient case volume per clinician, 52 clinicians each treated >15 patients, with a mean of 41.3 patients per clinician. In sum, this group of high-volume clinicians (2.6% of total clinicians) treated 44% of total study patients (Figure 1). The next 128 clinicians each treated 5–15 patients, while the remaining 1,824 clinicians treated 1–4 patients, most often with just a single patient (1,348). These clinician groups differed substantively in their median treatment duration per patient. The 52 clinicians with >15 patients had 6.3 median dispenses/patient (Figure 2). The 128 clinicians with 5–15 patients had 4.3 median dispenses; the interquartile range was 6.625–3.325. Treatment variability and early discontinuation increased with decreased patient volume; 1,348 clinicians with a single patient had two median dispenses, with 33% having only one dispense (*p* < 0.0001). Early discontinuation was significantly associated with lower patient volume (OR 0.80; CI 0.754–0.842). When early discontinuation occurred, it was often before a second dispense. Notably, the 80% of clinicians with one to four patients had a higher probability of early discontinuation that rarely occurred for clinicians with higher case volume of chlormethine gel.

## Discussion

Our findings emphasize that patients with MF-CTCL benefit from clinicians with more robust MF-CTCL experience and are in line with data reported by Kann et al. (6) showing that facilities with higher treatment volume, with as low as three patients, had improved overall survival in MF-CTCL and Sézary syndrome. Limitations of our analysis include defining treatment duration by dispense quantity, not by elapsed time, and that referral patterns may result in underestimation of treatment duration for some patients.

In conclusion, individual clinicians prescribing chlormethine gel for MF-CTCL varied considerably in patient volume and treatment duration. The small percentage (20%) of clinicians with higher patient volume (>5 patients) demonstrated longer treatment duration and avoided early discontinuation, potentially due to improved experience managing disease and treatment-associated dermatitis, and setting patient expectations. The early discontinuation noted among low-volume clinicians may identify lack of patient education on how to adhere to treatment. Seeking consultations from experienced clinicians may help to improve treatment outcomes.

## Data Availability Statement

The raw data supporting the conclusions of this article will be made available by the authors, without undue reservation.

## Ethics Statement

Ethical review and approval was not required for the study on human participants in accordance with the local legislation and institutional requirements. Written informed consent for participation was not required for this study in accordance with the national legislation and the institutional requirements.

## Author Contributions

CQ and GB contributed to conceptualization, design, and wrote the manuscript. CQ, TP, BH, GB, JA, and BP contributed to data collection and analysis. All authors critically revised and approved the final manuscript.

## Conflict of Interest

CQ has served as advisor/consultant for Helsinn, Miragen, Bioniz, Trillium, Kyowa Kirin; received research funding from Celgene. TP has served as advisor/consultant for Helsinn, Actelion. BH has served as advisor to Viracta Therapeutics. GB and JA are employees of Helsinn Therapeutics US Inc. BP has served as advisor/consultant for Bioniz, Helsinn, Kyowa Kirin, and Soligenix and as an investigator with grant support from Astex, Bioniz, Helsinn, Innate Pharma, Kyowa Kirin, Miragen, and Soligenix. Research funding was provided by Helsinn Therapeutics (U.S.), Inc., who were involved in: analysis plan, collection, management, data analysis and interpretation of the data, preparation, review, and approval of the manuscript.
